# Sex Differences in Response and Persistence to Biologic Therapy in Psoriatic Arthritis: A 52‐Week Analysis With Extended Long‐Term Outcomes

**DOI:** 10.1111/1346-8138.70108

**Published:** 2025-12-08

**Authors:** Keita Ohyachi, Saori Takamura, Kanade Iino, Souichiro Saito, Mizuki Takeuchi, Tomoo Fukuda

**Affiliations:** ^1^ Department of Dermatology Saitama Medical Center, Saitama Medical University Saitama Japan

**Keywords:** biologic therapy, propensity score‐adjusted analysis, psoriatic arthritis, sex differences, treatment persistence, treatment response

## Abstract

Psoriatic arthritis (PsA) is a chronic immune‐mediated disease with heterogeneous joint and skin manifestations. Although biologic therapies targeting TNFα, IL‐17, and IL‐23 have transformed PsA management, sex‐specific differences in efficacy and treatment persistence remain underexplored. Previous registry and real‐world data suggest that women may experience greater pain, lower treatment response, and shorter drug survival, but evidence integrating both articular and cutaneous outcomes is limited. This retrospective cohort study investigated whether female sex independently predicts reduced clinical response and persistence with biologic therapy in PsA, focusing on composite outcomes at week 52. A total of 134 patients (87 men, 47 women) who initiated biologics between 2011 and 2024 were analyzed. Diagnoses were confirmed according to CASPAR by board‐certified dermatologists, with rheumatologic confirmation when required. Propensity scores derived from baseline age, BMI, duration of psoriasis, duration of PsA, baseline Psoriasis Area and Severity Index (PASI), baseline Disease Activity index for Psoriatic Arthritis (DAPSA), comorbidities, NSAID use, and biologic class were incorporated into covariate‐adjusted ANCOVA models. At week 52, simultaneous achievement of PASI90 and DAPSA remission was observed in 51.2% of men versus 19.2% of women (*p* = 0.025). Female sex remained an independent negative predictor of response (adjusted OR = 0.19; 95% CI: 0.074–0.468; *p* = 0.0001). Kaplan–Meier analysis demonstrated shorter treatment persistence in women (log‐rank *p* = 0.0099; adjusted HR = 0.316, 95% CI: 0.119–0.841). Although only a few women (*n* = 3) cited financial burden as a reason for not re‐initiating biologics after discontinuation, this observation may still reflect underlying socioeconomic influences. Even small numbers of such cases can exemplify practical and economic barriers—often compounded by caregiving responsibilities or lower household income—that disproportionately affect women. Early and proactive intervention, prevention of unnecessary discontinuation, and timely re‐initiation upon relapse are essential to optimize outcomes in women with PsA.

## 1 Introduction

Psoriatic arthritis (PsA) is a chronic inflammatory disease requiring effective long‐term treatment to control joint and skin manifestations and improve quality of life [1, 2]. Biologic disease‐modifying therapies have revolutionized PsA management by achieving higher rates of remission and low disease activity than conventional agents. However, not all patients respond equally to these advanced therapies, and emerging evidence suggests that sex‐based differences may influence treatment outcomes. Female patients often present with a higher subjective disease burden than males, which can translate into differential therapeutic responses and persistence on treatment [3, 4, 5]. This raises clinically important questions: do women with PsA derive the same level of benefit from biologic treatments as men, and are their continuation rates on therapy comparable? Addressing these issues is crucial for optimizing PsA care, as any sex‐related disparities in outcomes might warrant tailored management strategies.

### 1.1 Sex‐Based Differences in Treatment Response

Retrospective studies have consistently found that male sex is associated with better treatment efficacy in PsA and related conditions. For example, an Italian cohort of psoriatic patients on anti–tumor necrosis factor (TNF)α therapy reported that men had over twice the odds of achieving a 75% improvement in disease severity (Psoriasis Area and Severity Index [PASI] 75) compared to women [6]. Likewise, in a large PsA registry, male patients showed significantly higher clinical response rates to TNF inhibitors than female patients. At 6 months of therapy, men were about three times more likely to attain a good treatment response (e.g., by ACR/EULAR criteria) than women (adjusted OR ~3.2) [7].

Analogous trends are seen in other populations. In a national psoriasis cohort, female sex was linked to a significantly lower likelihood of reaching near‐complete skin clearance under biologic therapy [8]. Similarly, in a real‐world analysis of inflammatory arthritis, women achieved remission on TNF blockers less frequently than men (45% vs. 59%) [9]. Collectively, these data indicate that female patients tend to experience somewhat lower clinical response rates to PsA treatments than their male counterparts.

### 1.2 Sex‐Based Differences in Treatment Persistence

Differences by sex also extend to treatment adherence and drug persistence. Multiple studies report that women discontinue biologic therapies sooner or at higher rates than men. In the Danish PsA registry, the median duration of first‐line TNF inhibitor therapy was 3.8 years in men versus only 1.4 years in women (*p* < 0.001) [7], illustrating substantially shorter drug survival in female patients. A similar gap was observed in a UK. PsA cohort, where female sex conferred approximately a 2.5‐fold higher risk of TNF inhibitor discontinuation compared to male sex (HR ~2.6) [10]. Consistently, a recent real‐world study identified female sex as an independent predictor of lower biologic persistence (i.e., higher likelihood of drug withdrawal) [11]. Notably, analogous patterns have been noted in psoriasis registries, in which female patients showed higher biologic discontinuation rates than males [12]. These findings suggest that, in practice, women with PsA are more prone to stop or switch therapy earlier, which may reflect differences in drug effectiveness, tolerability, or treatment expectations.

### 1.3 Hypothesized Reasons for Sex Disparities

The underlying reasons why women exhibit lower treatment responses and persistence are likely multifactorial. One hypothesis is that inherent immunological or hormonal differences play a role. Women generally mount more robust immune responses than men, potentially increasing the immunogenicity of biologic drugs and diminishing their efficacy. In theory, female patients might be more prone to developing anti‐drug antibodies that neutralize biologics. In practice, however, studies have not observed a significant sex difference in biologic drug levels or anti‐drug antibody formation in PsA [9], suggesting immunogenicity alone cannot explain the disparity.

Another contributing factor is that women with PsA often report greater pain, fatigue, and functional limitations than men at baseline [13, 14, 15]. Since composite outcome measures (e.g., ACR response criteria or minimal disease activity) include patient‐reported components, a higher symptom burden can result in women being less likely to meet stringent response or remission thresholds even when objective inflammation is similarly controlled. In other words, sex differences in pain perception and reporting may bias standard disease activity indices, making women appear to have poorer responses despite receiving effective therapy. However, this residual symptom burden does not necessarily translate into higher treatment persistence. In fact, patients who experience persistent pain or fatigue—despite adequate inflammatory control—may perceive limited benefit from continued biologic therapy, which can undermine motivation to maintain long‐term treatment. When coupled with socioeconomic and psychological factors such as financial constraints, caregiver responsibilities, or lower perceived treatment value, these factors may collectively contribute to the lower treatment continuation rates observed among women. Thus, sex‐related differences in both symptom perception and external barriers likely interact to shape treatment adherence and persistence in real‐world settings.

Behavioral and psychosocial factors related to treatment adherence may also contribute. Some evidence suggests that female patients are, on average, less satisfied with treatment outcomes and more cautious about side effects, which can affect their willingness to continue therapy [16, 17, 18]. Women may communicate medication concerns and adverse symptoms more readily and discontinue a drug if they feel it is not meeting their needs, whereas men might be more inclined to persist through moderate side effects or suboptimal improvement [7, 13, 18, 19]. For instance, certain adverse effects—such as cosmetic changes (e.g., hair loss) or weight gain—might prompt drug cessation more frequently in women due to greater psychosocial impact. Moreover, comorbid conditions like anxiety or depression are more prevalent in female PsA patients [7] and could influence their pain tolerance, perceptions of improvement, and persistence with treatment. These multifaceted factors, collectively, are thought to underlie the observed trend of women achieving lower treatment responses and shorter drug survival. Recognizing these sex‐based differences in PsA is important for clinicians and researchers alike, as it highlights the need for a sex‐sensitive approach to evaluating treatment outcomes and may inform more personalized management strategies.

In light of these considerations, the present study was designed to rigorously assess sex differences in PsA treatment evaluation—specifically, differences in clinical response to therapy and in treatment persistence—and to explore potential reasons for any observed disparities. By clarifying the extent and drivers of sex‐related differences in PsA outcomes, this research aims to contribute knowledge that can ultimately improve therapeutic decision‐making and patient care in PsA.

## 2 Subjects and Methods

### 2.1 Patient Population and Eligibility Criteria

This retrospective, single‐center observational cohort study was conducted at Saitama Medical Center, Saitama Medical University, Japan, between May 2011 and May 2024. The study adhered to the principles of the Declaration of Helsinki and was approved by the Institutional Review Board (Approval ID: 2025030). Given its retrospective design, patient consent was obtained using an opt‐out approach.

Adult patients (≥ 18 years) with PsA who initiated biologic therapy at our institution were eligible. Diagnoses were confirmed according to the Classification Criteria for Psoriatic Arthritis (CASPAR) by board‐certified dermatologists, with additional input from rheumatologists when necessary. Diagnostic confirmation was based on a comprehensive clinical evaluation, supported by musculoskeletal ultrasound and/or skin biopsy when indicated.

Exclusion criteria were as follows: (i) initiation of biologics at other institutions with incomplete follow‐up (*n* = 5); (ii) joint symptoms attributable to diseases other than PsA, such as rheumatoid arthritis or gout (*n* = 12); (iii) pregnancy or potential pregnancy (*n* = 1); and (iv) substantial missing data on key clinical variables, including Psoriasis Area and Severity Index (PASI), Disease Activity Index for Psoriatic Arthritis (DAPSA), or joint pain visual analog scale (VAS) (*n* = 6). After exclusions, 134 patients (87 men and 47 women) were included in the final analysis. Patient selection and exclusions are illustrated in Figure S1.

### 2.2 Data Collection and Variables

Baseline demographic and clinical data were extracted from electronic medical records at PsA diagnosis and biologic initiation, with follow‐up data collected at subsequent visits. Collected variables included:

*Demographics*: age, sex, height, weight, body mass index (BMI).

*Disease characteristics*: Age at psoriasis onset, age at PsA onset, duration of psoriasis, duration of PsA, and the interval between psoriasis and PsA onset.

*Comorbidities*: Hypertension, dyslipidemia, diabetes mellitus, and hyperuricemia.

*Treatment‐related factors*: Class of biologic therapy (TNF‐α, IL‐17, or IL‐23 inhibitor), prior biologic exposure, and concomitant nonsteroidal anti‐inflammatory drug (NSAID) use.

*Clinical outcomes*: PASI, DAPSA, VAS, reasons for discontinuation, re‐initiation of biologics, and occurrence of adverse events.

### 2.3 Outcome Measures

The primary outcome was the proportion of patients achieving the composite endpoint of PASI90 (≥ 90% reduction in PASI from baseline) and DAPSA remission (score < 4) at week 52, representing simultaneous control of skin and joint domains.

#### 2.3.1 Rationale for Selecting DAPSA Over MDA or CPDAI

DAPSA was selected as the joint‐domain outcome measure because it is a validated, continuous index that is highly sensitive to change and directly quantifies peripheral arthritis activity using components routinely collected in our clinical practice (tender and swollen joint counts, patient pain, patient global assessment, and CRP) [20]. This made it the most feasible and reliable instrument for retrospective evaluation.

In contrast, Minimal Disease Activity (MDA) and Composite Psoriatic Disease Activity Index (CPDAI) could not be applied consistently across the entire cohort. MDA requires fulfillment of 5 of 7 domains—including the Health Assessment Questionnaire (HAQ)—and HAQ data were missing for a subset of patients, precluding accurate post hoc calculation. Similarly, CPDAI could not be computed due to incomplete documentation of enthesitis scores and HAQ measures. Furthermore, because MDA is a stringent binary endpoint, it is less suitable for detecting gradations in treatment response in observational datasets compared with continuous measures such as DAPSA.

For these reasons, DAPSA was the most appropriate and analytically robust option for capturing joint disease activity in this retrospective study, particularly when paired with PASI90 to comprehensively evaluate skin and joint outcomes.

Secondary outcomes included:

Achievement rates of PASI75, 90, and 100 at weeks 16, 28, and 52.

Temporal changes in DAPSA and remission rates at weeks 16, 28, and 52.

Sex differences in baseline disease characteristics, including joint patterns and severity.

Drug survival and treatment continuation stratified by sex.

Reasons for discontinuation (inefficacy, adverse events, financial burden, patient preference).

Sex‐stratified treatment responses by biologic class.

Re‐initiation of biologics following discontinuation and outcomes after resumption.

The 52‐week time point was selected as the primary evaluation period because it reflects the long‐term, steady‐state efficacy and persistence of biologic therapy in routine clinical practice. While earlier time points (weeks 16 and 28) capture initial therapeutic response, week 52 allows assessment of sustained disease control across both joint and skin domains after stabilization of dosing and adherence patterns. Moreover, most real‐world registry studies and pivotal clinical trials in PsA adopt 52 weeks as a standard benchmark for evaluating durable treatment response and drug survival, facilitating meaningful comparison with prior literature and enhancing clinical interpretability of long‐term outcomes.

### 2.4 Clinical Assessment Tools

#### 2.4.1 Psoriasis Area and Severity Index

Calculated retrospectively from clinical documentation of erythema, scaling, and induration across body regions. PASI90 and PASI100 corresponded to ≥ 90% and 100% improvement from baseline, respectively.

#### 2.4.2 Disease Activity Index for Psoriatic Arthritis

Reconstructed from 66 swollen and 68 tender joint counts, C‐reactive protein (CRP) (mg/dL), patient global VAS, and pain VAS. Remission was defined as < 4.

#### 2.4.3 Pain VAS

Measured on a 0–100 mm scale, with higher scores indicating greater pain.

#### 2.4.4 Treatment Exposure and Categorization

Biologic therapies were classified into three groups:

- *TNF‐α inhibitors*: infliximab, adalimumab, certolizumab pegol.
- *IL‐17 inhibitors*: secukinumab, ixekizumab, brodalumab.
- *IL‐23 inhibitors*: guselkumab, risankizumab.

Biologic selection was at physician discretion, informed by disease characteristics, comorbidities, prior therapy, and insurance status. Treatment continuation was defined as uninterrupted biologic use until last follow‐up. Re‐initiation following discontinuation, whether with the same or a different biologic, was recorded as a new treatment episode.

### 2.5 Statistical Analysis

Continuous variables were presented as medians with interquartile ranges (IQRs), and categorical variables as frequencies with percentages. Between‐sex comparisons were performed using the Wilcoxon rank‐sum test for continuous variables and Fisher's exact test for categorical variables. Drug survival was assessed using Kaplan–Meier estimates and compared by log‐rank testing.

Propensity scores were generated using multivariable logistic regression, incorporating baseline age, BMI, duration of psoriasis, duration of PsA, baseline PASI, baseline DAPSA, comorbidities, NSAID use, and biologic class. Given residual imbalance and the risk of extreme weights, propensity score matching and inverse probability weighting were not used. Instead, the primary analysis applied a covariate‐adjusted ANCOVA model including the propensity score as a covariate.

All tests were two‐sided, with *p* < 0.05 considered statistically significant. Analyses were performed using JMP version 18 (SAS Institute, Cary, NC, USA) and SAS software version 9.4.

## 3 Results

### 3.1 Patient Characteristics

A total of 134 patients with PsA were included in the final analysis, comprising 87 men (64.9%) and 47 women (35.1%) (Table 1; Figure S1). The median age at study entry was 51 years (range: 26–74) in men and 53 years (range: 21–75) in women. Despite having lower absolute body weight, women exhibited a significantly higher median body mass index (BMI) compared with men (27.5 vs. 24.7 kg/m^2^, *p* < 0.05). The prevalence of comorbidities—including hypertension, dyslipidemia, diabetes mellitus, and hyperuricemia—was comparable between sexes, with no significant differences in distribution.

**TABLE 1:** Baseline demographic and clinical characteristics of patients with psoriatic arthritis stratified by sex.

Characteristic	Male (*N* = 87)	Female (*N* = 47)	*p*
Age (years)	51.0 (26.0–74.0)	53.0 (21.0–75.0)	0.1766
Age at PV onset (years)	39.0 (8.0–62.0)	43.0 (10.0–64.0)	0.1015
Age at PsA onset (years)	43.7 (8.0–73.5)	50.0 (15.0–74.0)	0.0252
Duration of PV (years)	10.0 (0.3–41.0)	7.0 (0.1–44.0)	0.1323
Interval between PV onset and PsA onset (years)	2.5 (−0.7–34.0)	4.0 (−4.0–37.0)	0.3678
Duration of PsA (years)	3.0 (0.1–38.0)	2.0 (0.1–19.0)	0.0249
Body weight (kg)	71.0 (53.5–115.0)	67.0 (35.0–86.0)	0.0032
Body Mass Index (kg/m^2^)	24.7 (18.7–41.0)	27.5 (15.6–39.3)	0.0311
Baseline Psoriasis Area and Severity Index	4.5 (0–54.0)	3.5 (0–39.2)	0.0815
Baseline DAPSA	20.5 (0.07–51.3)	19.1 (0.04–49.1)	0.3314
Pain VAS (mm)	60.0 (0–100)	50.0 (0–100)	0.3953
Type of arthritis			
Polyarthritis	58.6% (51)	51.1% (24)	0.4004
Oligoarthritis	34.5% (30)	44.7% (21)	0.2459
Spondylitis	2.3% (2)	0% (0)	0.2950
Combined spondylitis and polyarthritis	3.5% (3)	2.1 (1)	0.6682
Combined spondylitis and oligoarthritis	1.2% (1)	2.1 (1)	0.6558
Comorbidities			
Hypertension	11.5% (10)	10.6% (5)	0.8804
Dyslipidemia	27.6% (24)	29.8% (14)	0.7878
Diabetes mellitus	19.5% (17)	21.3% (10)	0.8116
Hyperuricemia	13.8% (12)	19.2% (9)	0.4211
Type of biologics			
TNF‐α	42.5% (37)	21.3% (10)	0.0139
IL‐17	49.4% (43)	48.9% (23)	0.9497
IL‐23	8.1% (7)	29.8% (14)	0.0010
NSAIDs	23.0% (20)	31.9% (15)	0.2659

*Note:* Data are presented as median (interquartile range [IQR]), range, or number.

Abbreviations: DAPSA, Disease Activity Index for Psoriatic Arthritis; IL, interleukin; NSAIDs, nonsteroidal anti‐inflammatory drugs; PsA, psoriatic arthritis; PV, psoriasis vulgaris; TNF, tumor necrosis factor; VAS, visual analog scale.

**FIGURE 1:**
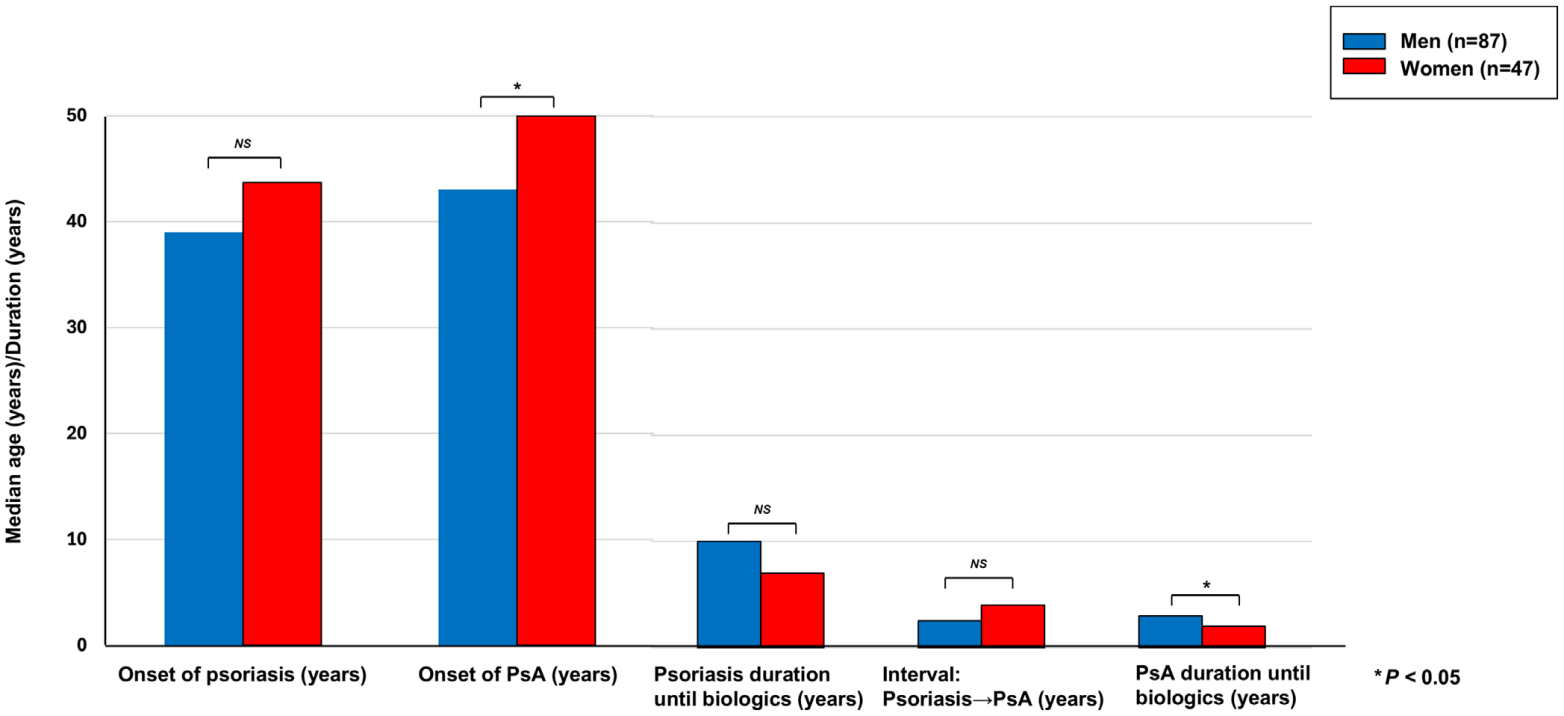
Clinical course of psoriasis and psoriatic arthritis (PsA) stratified by sex. Median age at psoriasis onset was 39.0 years in men and 43.0 years in women. PsA onset occurred later in women compared with men (50.0 vs. 43.7 years). The median interval from psoriasis onset to PsA onset was 4.0 years in women and 2.5 years in men. The duration of PsA prior to initiation of biologic therapy was shorter in women (2.0 vs. 3.0 years, *p* = 0.0249).

The clinical trajectory from psoriasis onset to PsA onset and initiation of biologic therapy revealed distinct sex‐related patterns (Figure 1). The median age at psoriasis onset was comparable between sexes (39.0 years in men vs. 43.0 years in women, *p* = n.s.), whereas the onset of PsA occurred significantly later in women (50.0 years) than in men (43.7 years, *p* < 0.05). Importantly, the median duration from PsA diagnosis to initiation of biologic therapy was significantly shorter in women than in men (2.0 vs. 3.0 years, *p* < 0.05). Given that baseline DAPSA scores were comparable between sexes (median: 20.5 in men vs. 19.1 in women), these findings suggest that female patients may undergo a more rapid escalation of disease activity following PsA onset, thereby necessitating earlier consideration of biologic intervention.

### 3.2 Patterns of Joint and Skin Involvement

Joint involvement patterns did not differ significantly between sexes (Table 1). Polyarthritis was the most common presentation (58.6% in men vs. 51.1% in women), followed by oligoarthritis (34.5% vs. 44.7%). Axial involvement and mixed forms were rare. Median pain visual analog scale (VAS) scores were similar (60 mm in men vs. 50 mm in women). For skin disease, men exhibited a trend toward higher median PASI scores (4.5 vs. 3.5), although the difference was not statistically significant.

### 3.3 Biologic Treatment Allocation

Significant sex‐related differences were observed in the choice of biologic therapy (Table 1). TNF‐α inhibitors were more frequently prescribed in men (42.5% vs. 21.3%), whereas IL‐23 inhibitors were administered more often in women (29.8% vs. 8.1%). The use of IL‐17 inhibitors was similar between sexes (49.4% vs. 48.9%). These findings highlight potential sex‐related preferences or clinical considerations influencing biologic selection.

### 3.4 Primary Outcome: Composite Response at Week 52

**FIGURE 2:**
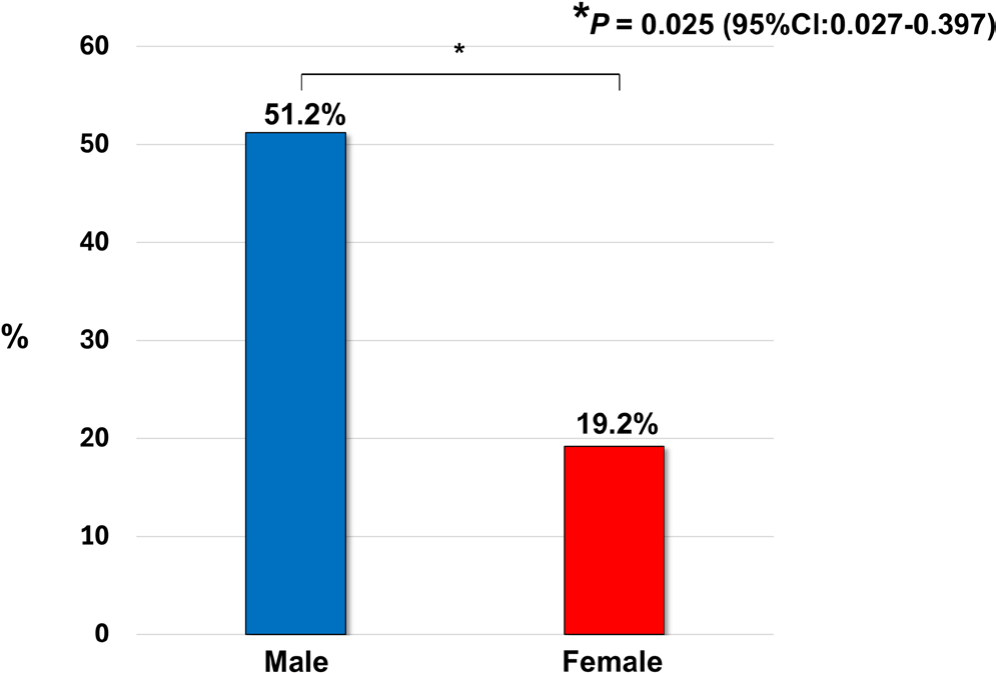
Achievement of the primary composite outcome at week 52. Proportion of patients achieving simultaneous Disease Activity in Psoriatic Arthritis (DAPSA) remission and the Psoriasis Area and Severity Index (PASI90) at Week 52, stratified by sex. Male patients achieved this endpoint significantly more often than female patients (51.2% vs. 19.2%, *p* = 0.025).

The primary outcome was defined as the simultaneous achievement of PASI90 and DAPSA remission at week 52. A significantly greater proportion of men achieved this stringent endpoint compared with women (51.2% vs. 19.2%, *p* = 0.025; 95% CI: 0.027–0.397) (Figure 2). In covariate‐adjusted analyses that accounted for baseline differences, female sex remained an independent negative predictor of treatment success, with an adjusted odds ratio of 0.19 (95% CI: 0.074–0.468; *p* = 0.0001) (Table 2).

**TABLE 2:** Multivariable logistic regression analysis of predictors of poor treatment response in patients with psoriatic arthritis.

	Adjusted model	
	OR (95% CI)	*p*
Sex (female)	0.19 (0.07–0.47)	0.0001
Age	1.03 (0.99–1.07)	0.1756
BMI	0.96 (0.88–1.05)	0.3402
Disease duration (psoriasis vulgaris)	0.99 (0.95–1.04)	0.8374
Disease duration (PsA)	1.07 (0.99–1.15)	0.0536
Baseline PASI	0.98 (0.94–1.03)	0.4679
Baseline DAPSA	0.99 (0.96–1.04)	0.9967

Abbreviations: BMI, body mass index; CI, confidence interval; DAPSA, Disease Activity Index for Psoriatic Arthritis; OR, odds ratio; PASI, Psoriasis Area and Severity Index; PsA, psoriatic arthritis.

To minimize potential residual confounding, propensity scores were incorporated as covariates into the analytic model. This approach allowed adjustment for baseline imbalances without the sample size loss and unstable estimates that can occur with propensity score matching or inverse probability weighting. Additionally, sensitivity analyses were conducted using logistic regression models that included each baseline variable individually. These analyses produced results consistent with the primary model, confirming that the observed association between female sex and reduced treatment efficacy was not driven by model dependency. Together, these analyses provide robust evidence that female sex independently predicts poorer composite outcomes in PsA.

### 3.5 Secondary Outcomes

#### 3.5.1 PASI Response

Sex‐related differences in cutaneous responses were evident across all time points. At week 16, and consistently thereafter, the proportions of patients achieving PASI75, PASI90, and PASI100 were numerically higher in men than in women, although these differences did not reach statistical significance (Table S1).

#### 3.5.2 DAPSA Remission

Similar patterns were observed in joint‐related outcomes. At week 16, DAPSA remission was achieved in 72.4% of men compared with 59.6% of women. By week 28, remission rates remained stable at 73.6% in men and 59.6% in women. At week 52, more than half of the male patients (71.3%) maintained remission, whereas only 61.7% of female patients achieved this outcome (*p* = 0.04; 95% CI: 0.10–0.35; Table S1).

#### 3.5.3 Extended Outcomes at Week 64

To further characterize longer‐term treatment trajectories, we additionally evaluated clinical outcomes at week 64 among patients who remained on biologic therapy. Although the number of evaluable patients decreased over time due to treatment switching or discontinuation, the week 64 dataset (men: 76; women: 42) still provided meaningful comparative insight into sustained therapeutic response (Table S1).

Consistent with the week 52 findings, women continued to show significantly lower efficacy across several skin and composite endpoints. Female patients achieved lower rates of PASI75 (47.6% vs. 75.0%, *p* = 0.003), PASI90 (42.9% vs. 65.8%, *p* = 0.02), and PASI100 (33.3% vs. 52.6%, *p* = 0.04) compared with men. Sex differences also persisted in composite outcomes integrating joint and skin responses, including DAPSA remission plus PASI75 (40.5% vs. 67.1%, *p* = 0.01) and DAPSA remission plus PASI90 (21.4% vs. 53.9%, *p* = 0.0004).

In contrast, DAPSA remission alone did not differ significantly between sexes (64.3% in women vs. 75.0% in men; *p* = 0.22), suggesting that the widening sex gap at 64 weeks was driven predominantly by lower skin response and difficulty attaining stringent composite targets rather than differences in joint‐only remission.

Taken together, these extended findings demonstrate that the sex disparities observed at week 52 persist beyond 1 year of therapy and, in some domains, become more pronounced by week 64. This reinforces the notion that female patients experience sustained challenges in achieving high‐level and composite treatment goals during long‐term biologic therapy. These longer‐term data directly address the reviewer's concern regarding the 52‐week window, demonstrating that the sex‐specific disparity in treatment response is not confined to the primary time point but remains evident—and in some cases widens—during extended follow‐up.

### 3.6 Sex‐Stratified Efficacy by Biologic Class

When stratified by biologic class, outcomes differed markedly by sex. Among TNF‐α inhibitor users, women exhibited substantially lower efficacy compared with men (3 of 10, 30.0% vs. 22 of 37, 59.5%). Similarly, responses to IL‐17 inhibitors were less favorable in women (6 of 23, 26.1%) than in men (22 of 43, 51.2%). In contrast, women treated with IL‐23 inhibitors achieved higher response rates than men (8 of 14, 57.1% vs. 2 of 7, 22.6%). These findings suggest that therapeutic outcomes may be differentially influenced by treatment class in a sex‐specific manner. However, given the limited sample sizes in each subgroup, these results should be interpreted with caution.

### 3.7 Treatment Persistence and Re‐Initiation

**FIGURE 3:**
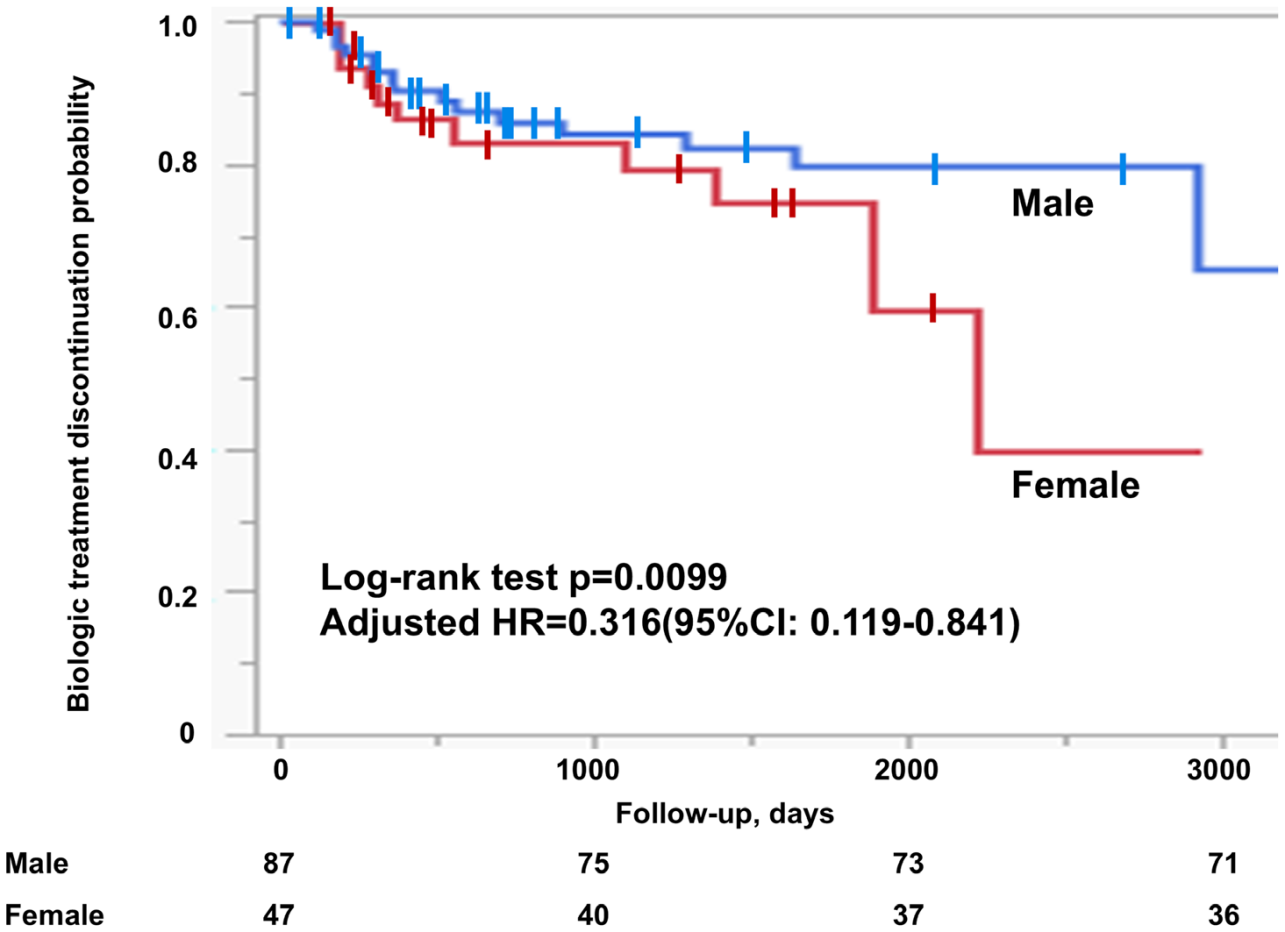
Proportion of biologic discontinuation stratified by sex in patients with psoriatic arthritis. Kaplan–Meier survival analysis demonstrated significantly reduced treatment continuation among women compared with men (log‐rank *p* = 0.0099), adjusted HR = 0.316 (95% CI: 0.119–0.841).

Kaplan–Meier survival analysis demonstrated significantly reduced treatment persistence in women compared with men (log‐rank test, *p* = 0.0099; adjusted HR = 0.316, 95% CI: 0.119–0.841) (Figure 3). In practical terms, although the adjusted hazard ratio is < 1 when drug continuation is modeled as the survival outcome, this corresponds to a substantially higher risk of treatment discontinuation among women. Using the reciprocal for interpretability, women had a roughly three‐fold higher risk of treatment discontinuation (HR ≈ 3.16) within 52 weeks compared with men.

At 1 year, 92.0% of men remained on biologic therapy, whereas only 87.2% of women did. By 5 years, this disparity widened further, with 83.9% of men still receiving therapy compared to 78.7% of women. Median follow‐up duration was significantly shorter in women than in men (870 days [range: 150–2920] vs. 1095 days [range: 120–5110], *p* = 0.0300).

The reasons for discontinuation of biologic therapy included secondary loss of efficacy, adverse events, financial burden, and patient preference (Table 3). Insufficient efficacy was reported more frequently among men, whereas adverse events and financial burden were more commonly cited by women. These observations underscore the influence of sex‐specific factors on long‐term treatment persistence, highlighting the importance of tolerability and socioeconomic barriers as critical determinants of therapeutic continuity. Such disparities emphasize the need for individualized management strategies that address both biological and socioeconomic challenges in sustaining biologic therapy among patients with PsA.

**TABLE 3:** Reasons for biologic therapy discontinuation stratified by sex in patients with psoriatic arthritis.

Reason for discontinuation	Male (*N* = 16)	Female (*N* = 11)
Secondary loss of efficacy	68.8% (11)	18.2% (2)
Adverse events	6.3% (1)	27.3% (3)
Financial burden	6.3% (1)	27.3% (3)
Patient preference	18.3% (3)	27.3% (3)

*Note:* Data are presented as median (interquartile range [IQR]), range, or number.

**FIGURE 4:**
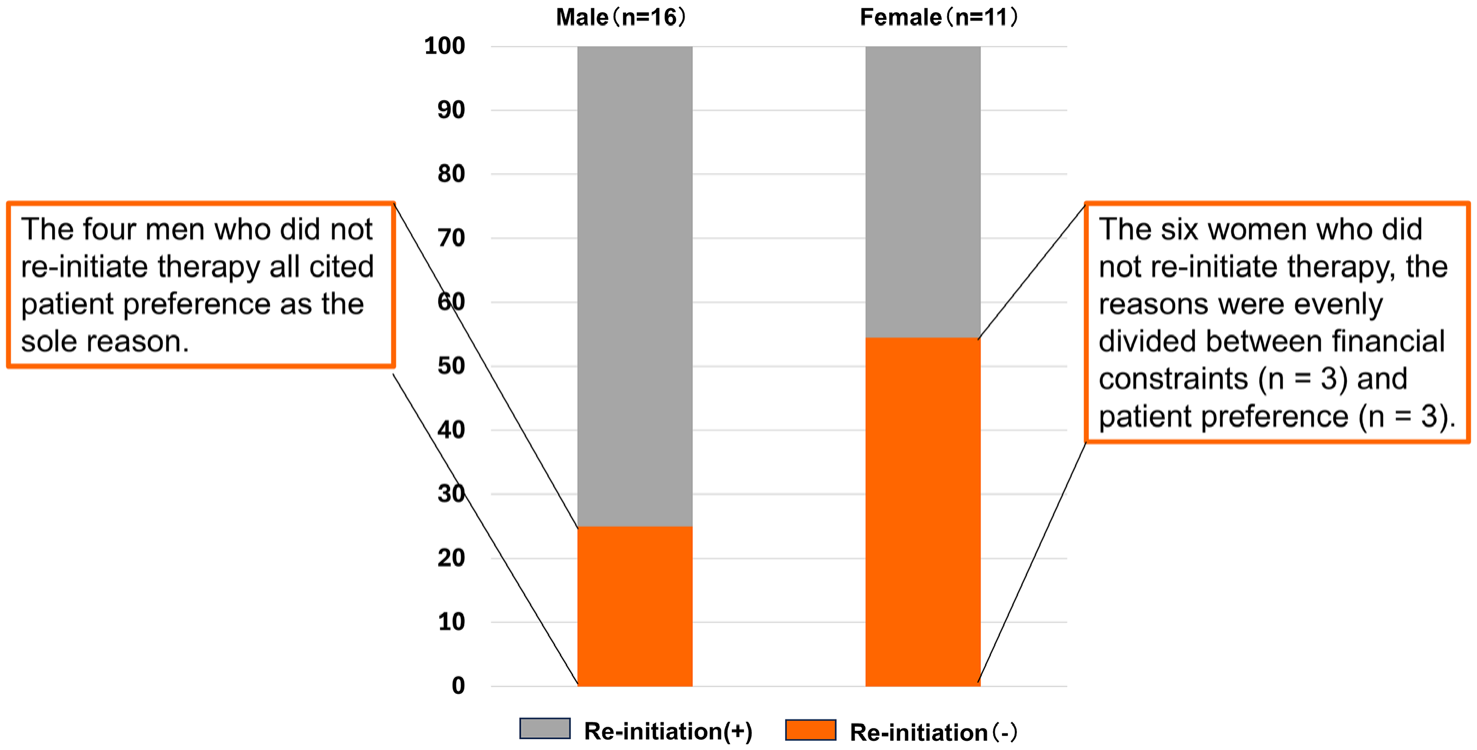
Sex‐based differences in the re‐initiation of biologic therapy following discontinuation in patients with psoriatic arthritis. Re‐initiation of biologic therapy—defined as restarting either the same or a different biologic after treatment discontinuation due to recurrence of joint symptoms—occurred in 75.0% of men (12/16) compared with 45.5% of women (5/11). Although this difference did not reach statistical significance (*p* = 0.118), the numerical disparity suggests that women were less likely to resume biologic therapy after discontinuation. Among the six women who did not re‐initiate therapy, the reasons were evenly divided between financial constraints (*n* = 3) and patient preference (*n* = 3), whereas all four men who did not re‐initiate cited patient preference alone.

Re‐initiation of biologic therapy—defined as restarting either the same or a different biologic agent following treatment discontinuation due to recurrence of articular symptoms—was more common among men (75.0%) than women (45.5%) (Figure 4). Although this difference did not reach statistical significance, the numerical disparity suggests that women who discontinue biologic therapy are less likely to resume treatment, potentially exacerbating disease control challenges over time (*p* = 0.118). Importantly, the underlying reasons for non–re‐initiation differed by sex: all four men who did not resume biologic therapy cited patient preference as the sole reason, whereas the six women who did not re‐initiate therapy reported financial constraints (*n* = 3) and patient preference (*n* = 3) in equal proportions.

### 3.8 Adverse Events

Adverse events were documented in 8 men (9.2%) and 7 women (14.9%) during the observation period (Table 4). Reported events included infections such as cellulitis and oral candidiasis, dermatologic complications including eczema and folliculitis, and isolated systemic effects such as elevations in liver enzymes. No statistically significant sex‐based differences were observed in the overall frequency of adverse events.

**TABLE 4:** Adverse events by sex in patients with psoriatic arthritis treated with biologic therapies.

Adverse events	Male (*N* = 87)	Female (*N* = 47)	*p*
Elevated AST/ALT	0% (0)	2.1% (1)	0.1721
Acne/Furuncle	1.1% (1)	4.3% (2)	0.2462
Oral candidiasis	1.1% (1)	2.1% (1)	0.6558
Tinea pedis	1.1% (1)	0% (0)	0.4607
Eyelid dermatitis	1.1% (1)	2.1% (1)	0.3142
Glossitis	0% (0)	2.1% (1)	0.1721
Cellulitis	1.1% (1)	0% (0)	0.4607
Necrotizing fasciitis	1.1% (1)	0% (0)	0.4607
Purulent knee arthritis	1.1% (1)	0% (0)	0.4607
Eczema	1.1% (1)	2.1% (1)	0.6558
Total adverse events	9.2% (8)	14.9% (7)	0.3181

*Note:* Data are presented as median (interquartile range [IQR]), range, or number.

Abbreviation: AST/ALT, aspartate aminotransferase/alanine aminotransferase.

Serious adverse events were rare. Necrotizing fasciitis and septic arthritis were each reported as isolated cases, and their occurrence did not differ between sexes. Importantly, no treatment‐related deaths were recorded.

## 4 Discussion

This study provides new evidence that sex‐specific differences significantly impact biologic therapy outcomes in PsA. We observed that at 52 weeks of treatment, female patients had lower clinical response rates and poorer drug persistence compared to male patients. Notably, women were significantly less likely to achieve the combined target of DAPSA remission (for arthritis) together with PASI90 skin clearance, even after adjusting for baseline disease characteristics via propensity scoring. This finding underscores a reduced treatment efficacy in women that is not explained by confounding factors. In addition, treatment persistence on biologic therapy was markedly lower in women. Kaplan–Meier analysis demonstrated that women discontinued biologics at higher rates and earlier time points than men. This sex gap in drug survival suggests that women not only respond less optimally but also have greater difficulty maintaining long‐term therapy. Following discontinuation, women were also less likely to re‐initiate biologic treatment after disease relapse. Although the number of cases citing financial burden as the reason for non–re‐initiation was small (*n* = 3), this observation may still reflect an underlying socioeconomic influence. In real‐world clinical settings, even a few cases can exemplify the financial and logistical barriers that disproportionately affect women, particularly when compounded by caregiving responsibilities or lower household income. Therefore, while these findings should be interpreted with caution due to the limited sample size, they underscore the importance of addressing financial accessibility and social support as part of comprehensive, sex‐conscious PsA management strategies. We also found an intriguing pattern in disease course: on average, women in our cohort developed PsA at a later age than men, yet they progressed to requiring a biologic more quickly after onset. This suggests that once PsA manifests in females, it may reach a level warranting advanced therapy more rapidly, hinting at a potentially more aggressive disease trajectory or a lower threshold for escalation in women. In addition to the 52‐week primary endpoint, we further examined longer‐term outcomes at Week 64 in patients who remained on biologic therapy. These extended follow‐up data demonstrated that the sex‐specific differences observed at 52 weeks persisted beyond 1 year: women continued to show significantly lower achievement rates for PASI75, PASI90, PASI100, and combined joint–skin endpoints (DAPSA remission + PASI75 or PASI90). Although the number of patients decreased due to discontinuation or switching, the overall pattern remained consistent, supporting the robustness and durability of the observed sex disparities. These findings reinforce that female sex is associated not only with reduced early treatment response but also with persistently lower long‐term efficacy, thereby validating the use of the 52‐week time point as a clinically meaningful benchmark aligned with existing PsA trials and registry data.

Taken together, these findings indicate that female sex predicts a more difficult treatment path in PsA, with diminished likelihood of reaching remission and shorter persistence on effective therapies by 1 year. These disparities are clinically meaningful and merit in‐depth exploration.

In addition to these findings, we observed a notable sex difference in the selection of biologic classes. Women more frequently received IL‐23 inhibitors, whereas TNF‐α inhibitors were more common in men. This pattern may partly reflect temporal factors, as IL‐23 inhibitors were introduced during the later years of the study period, and women in our cohort tended to initiate biologic therapy slightly later than men. It is also possible that treatment choice was influenced by clinical or practical considerations, such as the strong cutaneous efficacy and convenient dosing schedule of IL‐23 inhibitors, which may be particularly appealing to female patients with high skin‐related quality‐of‐life burdens. Conversely, TNF‐α inhibitors have been available for a longer period and may have been more commonly used among male patients who started therapy earlier in the treatment era. Although our study cannot definitively determine causality, these observations suggest that both temporal availability and patient‐ or physician‐driven preferences may contribute to the sex‐specific patterns of biologic use. Further research is warranted to clarify whether biological, psychosocial, or contextual factors underlie these treatment allocation differences.

Our results align with a growing body of literature identifying sex‐based disparities in PsA outcomes. In particular, they corroborate findings from large real‐world registries and cohorts. For example, an analysis from the Danish *DANBIO* registry (*n* ≈ 1750) reported that male PsA patients had significantly better responses to TNF inhibitors and longer drug survival than females [7]. In that study, the median TNFi persistence was 3.8 years in men versus only 1.4 years in women, and male sex conferred over a three‐fold higher odds of achieving a good clinical response at 6 months (adjusted OR ~3.2) compared to female sex, despite adjustment for baseline disease activity and comorbidities. Similarly, the recent multicenter *PsABio* study compared biologic outcomes in male and female PsA patients starting either a TNF inhibitor or ustekinumab. After 12 months of therapy, a significantly greater proportion of men had reached low disease activity or minimal disease activity, whereas women lagged behind on all key measures [21]. For instance, nearly 80% of men achieved low disease activity at 1 year in PsABio, compared to ~58% of women, and women had persistently worse patient‐reported outcomes alongside higher discontinuation rates. These real‐world data mirror our findings at 52 weeks, reinforcing the notion that female PsA patients tend to have less favorable treatment trajectories on biologic DMARDs. Beyond TNF inhibitors, a recent U.S. cohort study of PsA patients noted that female sex was associated with more than double the odds of requiring multiple biologic or targeted synthetic DMARDs (indicative of *difficult‐to‐treat* disease), along with factors like obesity and depression [22]. Thus, across diverse populations and treatment eras, female PsA patients emerge as a subgroup achieving remission less often and switching therapies more frequently than their male counterparts. This pattern is reminiscent of observations in other inflammatory arthritides (such as rheumatoid arthritis), highlighting sex as a fundamental determinant of treatment outcome in immune‐mediated disease.

The etiology of these sex disparities is likely multifactorial, involving a complex interplay of biological, pharmacokinetic, and psychosocial factors. Biologically, sex‐related differences in immune function and disease phenotype could contribute to differential responses. Females generally mount more robust humoral and cell‐mediated immune responses, which, while advantageous for pathogen defense, can translate into a propensity for hyperimmune inflammation and autoimmunity. Estrogens have been shown to stimulate certain pro‐inflammatory pathways, whereas androgens may be immunomodulatory; this hormonal milieu might make inflammatory arthritis relatively more refractory in women [23, 24]. It is also possible that women's immune systems generate anti‐drug antibodies or clear biologic agents at different rates, resulting in reduced drug bioavailability over time, although direct evidence in PsA is limited. Apart from immunology, sex differences in disease presentation may play a role. Prior studies indicate that men and women do not exhibit identical PsA phenotypes: for example, male patients have been reported to have more severe skin psoriasis and a higher tendency toward radiographic joint damage, whereas female patients more often report diffuse pain, fatigue, and functional limitations out of proportion to objective findings [13, 14, 15]. In our cohort, women had to achieve both joint remission and high‐level skin improvement (PASI90) to count as treatment success, and failing either component would deem the outcome suboptimal. If female patients tended to have, say, more extensive joint pain or enthesitis that is harder to fully quell, this could lower their odds of meeting a stringent composite endpoint. Conversely, if male patients generally start with more severe skin involvement, they may experience dramatic visible improvements on therapy (e.g., high PASI response), boosting their overall success rate. In short, inherent differences in disease burden and domains affected (skin vs. joints vs. pain) by sex can influence who attains comprehensive remission criteria.

Psychosocial and behavioral factors are also important in interpreting our findings. A consistent observation across PsA cohorts is that women report higher baseline pain and a greater subjective disease impact than men, even when objective inflammation (swollen joint counts, CRP, etc.) is similar [13, 14, 15]. In our study, despite receiving comparable biologic treatments, women were less likely to reach states like DAPSA remission which rely partly on patient‐reported outcomes. This suggests that women's higher pain sensitivity and symptom reporting may impede them from being classified in remission, a phenomenon also noted in rheumatoid arthritis studies. Women with PsA have nearly twice the prevalence of mood disorders such as anxiety and depression compared to men in some registries [7], and this psychological burden can amplify pain perception and reduce the perceived effectiveness of treatments. It is notable that in a prospective early PsA cohort, after 1 year of therapy, female patients still had higher pain scores, worse function, and lower quality of life than male patients, indicating they did not “catch up” despite similar treatment protocols. Investigators postulated that some women may have been undertreated or that standard measures were not capturing their disease adequately, raising concern that a one‐size‐fits‐all approach might be leaving female patients with ongoing symptoms. Additionally, medication tolerability may differ by sex: our data and others' suggest women are more prone to report side effects and discomfort with therapies. In an early PsA study, 58% of medication side‐effect reports came from women (vs 42% from men), and women tended to discontinue certain drugs sooner, possibly owing to lower tolerance. This higher rate of adverse effect reporting could lead to more frequent drug stops or switches in women, contributing to the lower persistence we observed. Thus, sex‐specific differences in pain threshold, mental health, and drug tolerability are likely pivotal contributors to the reduced response and persistence in female PsA patients.

Another novel aspect highlighted by our study is the impact of socioeconomic factors and patient priorities on treatment continuation, which appeared to disadvantage women. Financial constraints were cited disproportionately by female patients who stopped biologics and did not resume therapy. This finding resonates with broader societal trends: women, on average, may have less financial independence or are more often in part‐time employment with inferior health insurance coverage. In support of this, a large cohort in early PsA found that only 64% of women were in paid employment, compared to 78% of men, and women had slightly lower educational attainment on average [7, 13, 18, 19]. Such factors can translate into greater difficulty affording expensive biologic medications or navigating insurance approvals, leading to cost‐driven non‐adherence. Moreover, women often juggle caregiving roles (for children or elderly family) which might make them less likely to prioritize their own long‐term treatment or to attend frequent injection/infusion appointments. Some women of childbearing age may also elect to discontinue or avoid biologics due to concerns about medication effects on pregnancy and fertility. These practical and social considerations could explain why, upon experiencing a disease flare or relapse, female patients in our study were reluctant to re‐initiate therapy. It underscores that effective disease management must go beyond drug selection and address real‐world barriers. Proactively offering financial counseling, facilitating access to patient assistance programs, and scheduling flexible treatment regimens (e.g., home injections or aligning therapy timing with family plans) may particularly benefit female patients and improve their persistence on therapy.

Our observation that women had a later PsA onset but a shorter interval from diagnosis to biologic initiation also deserves discussion. One interpretation is that disease progression might be accelerated in women once PsA manifests. Perhaps women, who tend to develop PsA at an older age in our cohort, experience a more explosive course requiring rapid escalation to advanced therapies. This could be related to post‐menopausal immune changes or other age‐related factors that make PsA in older women especially active. Another consideration is whether there has been a shift in clinical practice toward *earlier aggressive treatment* in women, precisely because studies have highlighted their poorer outcomes. It is interesting to contrast our finding with data from a Dutch early PsA cohort, which noted a non‐significant trend toward delayed initiation of biologics in women relative to men in the first year of disease. Historically, there may have been physician bias or other barriers resulting in women being started on biologics later. Our contemporary cohort, however, suggests that when women present with PsA, they are now being escalated promptly—possibly reflecting better recognition of their high disease burden or patient self‐advocacy for more effective treatment. These nuances indicate that sex differences in treatment timelines can vary, and they warrant further study. Regardless of the cause, the clinical message is that we must ensure *timely intervention* in female PsA patients. Given their propensity for rapid escalation of therapy, careful monitoring early in the disease course is important so that effective treatments (including biologics) are introduced at the optimal time to prevent irreversible joint damage.

The implications of our findings are considerable for both research and clinical practice. From an academic perspective, this study reinforces that sex is a critical factor influencing treatment response in PsA, and future studies should systematically incorporate sex‐stratified analyses when evaluating long‐term outcomes. It will be important to clarify whether observed differences reflect biological, psychosocial, or treatment‐related factors, and to determine how these may be addressed through optimized therapeutic strategies. Such investigations could ultimately contribute to the development of individualized, equitable treatment approaches for patients with PsA.

Clinically, our results highlight the importance of tailoring PsA management with attention to sex‐specific differences. Practitioners should recognize that a female PsA patient who is not meeting treatment targets may not simply be a “difficult” case in isolation, but part of a broader pattern necessitating proactive management. This could include ensuring tight *treat‐to‐target* monitoring for women, with readiness to adjust therapy more quickly if low disease activity is not attained. Combination therapy or adjuncts might be considered—for example, concurrent fibromyalgia management, physical therapy for pain, or psychological support—to address dimensions of disease that predominantly affect women. It is also crucial to engage female patients in shared decision‐making that acknowledges concerns like family planning or work‐life balance, thereby improving trust and adherence. Finally, health systems and providers should strive to mitigate financial barriers by guiding patients to support programs or advocating for insurance coverage, since improving access and persistence will directly translate to better outcomes.

Despite these important insights, this study has several limitations that should be acknowledged. First, the retrospective, single‐center design may limit generalizability, and unmeasured confounding factors—such as disease phenotype, pain sensitization, or psychosocial variables—cannot be completely excluded. Second, treatment selection was not randomized and was influenced by physician discretion, which may have introduced indication bias despite adjustment with propensity scores. Third, the sample size within each biologic subclass (particularly IL‐23 inhibitors) was relatively small, restricting the statistical power for subgroup analyses and limiting the interpretation of class‐specific effects. Finally, treatment persistence and re‐initiation data were obtained from real‐world clinical records, and external factors such as socioeconomic status or insurance coverage were not formally quantified. Nevertheless, the consistency of findings across multiple sensitivity analyses and the integration of both efficacy and persistence outcomes strengthen the reliability and clinical relevance of our conclusions.

In conclusion, our study's findings emphasize that a sex‐sensitive approach—one that integrates awareness of biological, psychosocial, and economic factors differentiating male and female patients—is essential to optimize PsA care. By tailoring treatment strategies in this way, we can move closer to equitable disease control and improve the long‐term prognosis for all patients with PsA, women and men alike.

## Funding

The authors have nothing to report.

## Conflicts of Interest

S.T. has received lecture fees from Torii Pharmaceutical, Takeda Pharmaceutical, CSL Behring, Abbvie, UCB Japan, Janssen Pharmaceutical, Taiho Pharmaceutical, Maruho, Novartis Pharma, Kyowa Hakko Kirin, Eli Lilly, LEO Pharma, and Sanofi. T.F. has received lecture fees from Sato Pharmaceutical, Eli Lilly, Abbvie, and CSL Behring. Drs. Ohyachi, Iino, Saitou, and Takeuchi declare no conflicts of interest.

## Supporting information


**Figure S1:** Flowchart of patient selection for study participants. Among 158 patients initially screened, 24 were excluded due to initiation of biologic therapy at other institutions with incomplete follow‐up data (*n* = 5), joint manifestations attributable to non‐psoriatic arthritis (PsA) conditions such as rheumatoid arthritis or gout (*n* = 12), pregnancy (*n* = 1), or substantial missing clinical data including Psoriasis Area and Severity Index (PASI), Disease Activity index for Psoriatic Arthritis (DAPSA), or joint pain visual analog scale (VAS) (*n* = 6). Ultimately, 134 patients who fulfilled the Classification Criteria for Psoriatic Arthritis (CASPAR) criteria and had complete clinical data for both joint and skin manifestations were included in the final analysis.

**Table S1:** Primary and secondary clinical outcomes by sex at weeks 16, 28, 52, and 64. Table S1 summarizes sex‐stratified analyses of clinical outcomes in patients with psoriatic arthritis treated with biologic agents. At weeks 16, 28, and 52, women consistently demonstrated lower response rates than men across multiple efficacy endpoints, including PASI75, PASI90, and composite outcomes incorporating DAPSA remission. The greatest disparity at week 52 was observed for the composite endpoint of DAPSA remission plus PASI90, achieved by 19.2% of women compared with 51.2% of men (*p* = 0.03). To complement these findings, long‐term outcomes at week 64 were additionally evaluated among patients who remained on biologic therapy. Sex differences persisted, with women showing significantly lower achievement rates for PASI75, PASI90, PASI100, and the composite outcomes incorporating DAPSA remission plus PASI75 or PASI90. These results indicate that the sex‐related disparity in treatment response was not limited to the 52‐week time point but extended into longer‐term follow‐up.

## Data Availability

Data sharing not applicable to this article as no datasets were generated or analyzed during the current study.
