# Supplementary material for: Sex Differences in Response and Persistence to Biologic Therapy in Psoriatic Arthritis: A 52‐Week Analysis With Extended Long‐Term Outcomes
Source: J Dermatol. 2025 Dec 8;53(2):219–30. doi: 10.1111/1346-8138.70108 (PMC12877971; doi:10.1111/1346-8138.70108)
Supplement: Supplementary file 1 — Figure S1: Flowchart of patient selection for study participants. Among 158 patients initially screened, 24 were excluded due to initiation of biologic therapy at other institutions with incomplete follow‐up data (n = 5), joint manifestations attributable to non‐psoriatic arthritis (PsA) conditions such as rheumatoid arthritis or gout (n = 12), pregnancy (n = 1), or substantial missing clinical data including Psoriasis Area and Severity Index (PASI), Disease Activity index for Psoriatic Arthritis (DAPSA), or joint pain visual analog scale (VAS) (n = 6). Ultimately, 134 patients who fulfilled the Classification Criteria for Psoriatic Arthritis (CASPAR) criteria and had complete clinical data for both joint and skin manifestations were included in the final analysis. [file JDE-53-219-s002.docx]

**Supplementary Table 1. Secondary Clinical Outcomes by Sex at Weeks 16, 28, and 52.**

| **Secondary Clinical Outcomes** | **Male (N = 87)** | **Female (N = 47)** | **P value (95%CI)** |
| --- | --- | --- | --- |
| **Week16** |  |  |  |
| **DAPSA remission** | 72.4% (63) | 59.6% (28) | 0.22 (0.05–0.25) |
| **PASI75** | 78.2% (68) | 55.3% (26) | 0.72 (-0.13–0.19) |
| **PASI90** | 59.8% (52) | 40.4% (19) | 0.79 (-0.14–0.18) |
| **PASI100** | 36.8% (32) | 36.2% (17) | 0.88 (-0.12–0.14) |
| **DAPSA remission+PASI75** | 57.5% (50) | 38.3% (18) | 0.09 (0.03–0.37) |
| **DAPSA remission+PASI90** | 43.7% (38) | 27.7% (13) | 0.13 (0.04–0.34) |
| **DAPSA remission+PASI100** | 29.9% (26) | 25.5% (12) | 0.49 (-0.12–0.25) |
| **Week28** |  |  |  |
| **DAPSA remission** | 73.6% (64) | 59.6% (28) | 0.07 (-0.02–0.32) |
| **PASI75** | 79.3% (69) | 53.2% (25) | 0.54 (-0.11–0.21) |
| **PASI90** | 57.5% (50) | 42.6% (20) | 0.91 (-0.16–0.18) |
| **PASI100** | 43.7% (38) | 36.2% (17) | 0.66 (-0.11–0.17) |
| **DAPSA remission+PASI75** | 60.9% (53) | 40.3% (19) | 0.06 (-0.04–0.39) |
| **DAPSA remission+PASI90** | 42.5% (37) | 31.9% (15) | 0.34 (-0.10–0.29) |
| **DAPSA remission+PASI100** | 35.6% (31) | 29.8% (14) | 0.29 (-0.87–0.29) |
| **Week52** |  |  |  |
| **DAPSA remission** | 71.3% (62) | 61.7% (29) | 0.04 (0.10–0.35) |
| **PASI75** | 72.4% (63) | 48.9% (23) | 0.57 (-0.1–0.18) |
| **PASI90** | 59.8% (52) | 42.6% (20) | 0.71 (-0.13–0.19) |
| **PASI100** | 47.1% (41) | 34.0% (16) | 0.78 (-0.12–0.16) |
| **DAPSA remission+PASI75** | 63.2% (55) | 40.4% (19) | 0.14 (-0.05–0.34) |
| **DAPSA remission+PASI90** | 51.2% (45) | 19.2% (9) | 0.03 (0.03–0.40) |
| **DAPSA remission+PASI100** | 42.5% (37) | 29.8% (14) | 0.18 (-0.06–0.33) |
| **Week64** |  |  |  |
| **DAPSA remission** | 75.0% (57/76) | 64.3% (27/42) | 0.22 (-0.07–0.28) |
| **PASI75** | 75.0% (57/76) | 47.6% (20/42) | 0.003 (0.10–0.45) |
| **PASI90** | 65.8% (50/76) | 42.9% (18/42) | 0.02 (0.01–0.41) |
| **PASI100** | 52.6% (40/76) | 33.3% (14/42) | 0.04 (0.01–0.37) |
| **DAPSA remission+PASI75** | 67.1% (51/76) | 40.5% (17/42) | 0.01 (0.10–0.45) |
| **DAPSA remission+PASI90** | 53.9% (41/76) | 21.4% (9/42) | 0.0004 (0.16–0.49) |
| **DAPSA remission+PASI100** | 44.7% (34/76) | 31.0% (13/42) | 0.14 (⊟0.04–0.32) |

Note. Data are presented as median (interquartile range [IQR]), range, or number.

Abbreviations: DAPSA, Disease Activity Index for Psoriatic Arthritis; PASI, Psoriasis Area and Severity Index
