# Supplementary figures and images for: Sex Differences in Response and Persistence to Biologic Therapy in Psoriatic Arthritis: A 52‐Week Analysis With Extended Long‐Term Outcomes
Source: J Dermatol. 2025 Dec 8;53(2):219–30. doi: 10.1111/1346-8138.70108 (PMC12877971; doi:10.1111/1346-8138.70108)

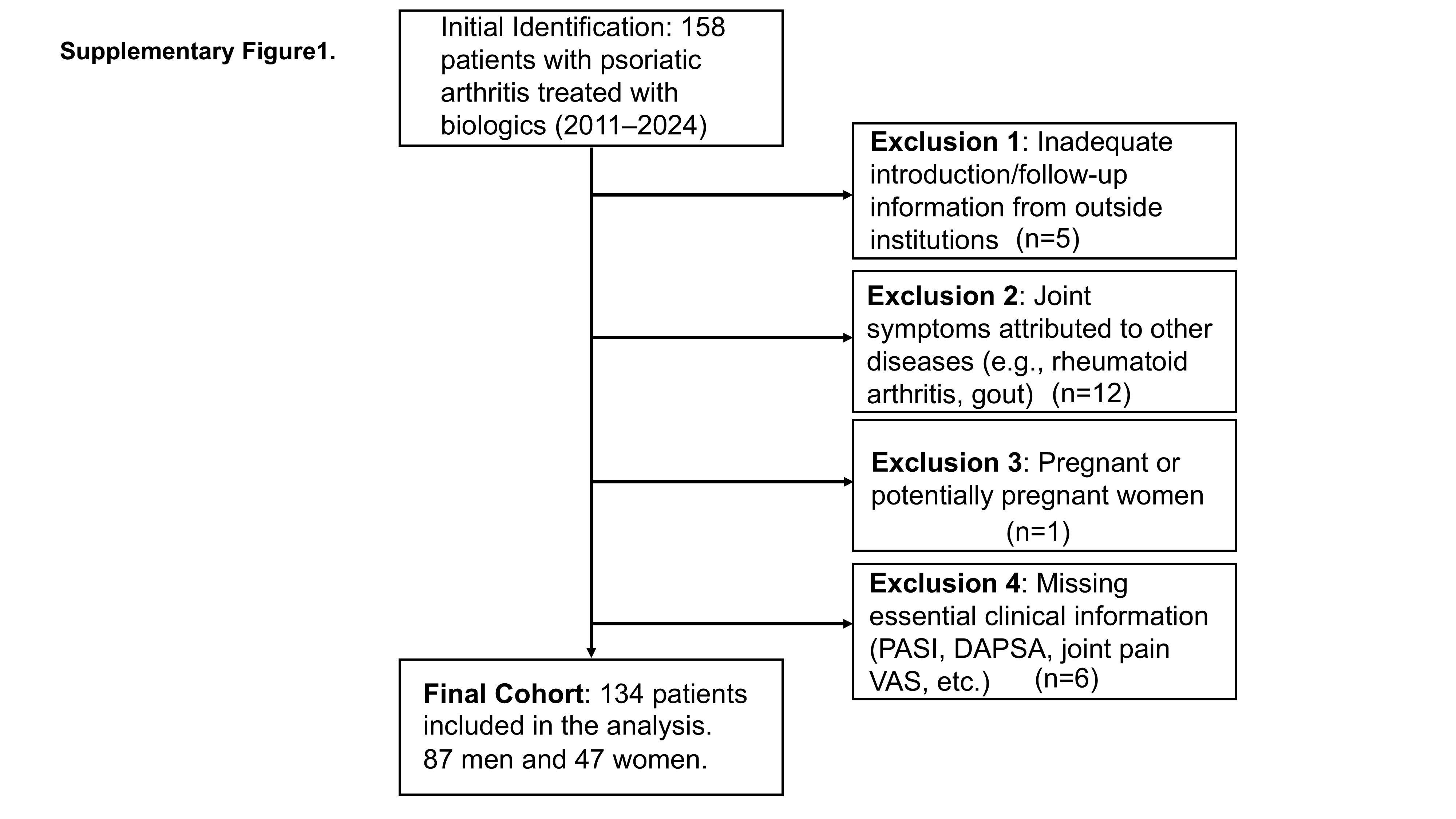

Supplement: Supplementary file 2 — Table S1: Primary and secondary clinical outcomes by sex at weeks 16, 28, 52, and 64. Table S1 summarizes sex‐stratified analyses of clinical outcomes in patients with psoriatic arthritis treated with biologic agents. At weeks 16, 28, and 52, women consistently demonstrated lower response rates than men across multiple efficacy endpoints, including PASI75, PASI90, and composite outcomes incorporating DAPSA remission. The greatest disparity at week 52 was observed for the composite endpoint of DAPSA remission plus PASI90, achieved by 19.2% of women compared with 51.2% of men (p = 0.03). To complement these findings, long‐term outcomes at week 64 were additionally evaluated among patients who remained on biologic therapy. Sex differences persisted, with women showing significantly lower achievement rates for PASI75, PASI90, PASI100, and the composite outcomes incorporating DAPSA remission plus PASI75 or PASI90. These results indicate that the sex‐related disparity in treatment response was not limited to the 52‐week time point but extended into longer‐term follow‐up. [file JDE-53-219-s001.tif]
